# Bio‐ethical issues in oncology during the first wave of the COVID‐19 epidemic: A qualitative study in a French hospital

**DOI:** 10.1111/jep.13766

**Published:** 2022-09-15

**Authors:** Henri‐Corto Stoeklé, Laure Ladrat, Terence Landrin, Philippe Beuzeboc, Christian Hervé

**Affiliations:** ^1^ Department of Ethics and Scientific Integrity Foch Hospital Suresnes France; ^2^ Department of Oncology and Supportive Care Foch Hospital Suresnes France; ^3^ Department of Supportive Care Cognacq‐Jay Hospital Paris France; ^4^ Medical School Paris Cité University Paris France; ^5^ Medical School Versailles‐Saint‐Quentin‐en‐Yvelines University (UVSQ) Montigny‐le‐Bretonneux France; ^6^ International Academy of Medical Ethics and Public Health Paris Cité University Paris France; ^7^ Veterinary Academy of France Paris France

**Keywords:** bioethicist, bioethics, cancer patient, COVID‐19, first wave, hospitals, oncologist, oncology

## Abstract

**Background:**

Using a specific bioethical theory (=global bioethics) and method (=a posteriori), we try here to identify and evaluate the bio‐ethical issues raised by the COVID‐19 pandemic, and possible solutions, to improve the management of cancer patients at the hospital in future pandemics, before the emergence of vaccines or scientifically validated treatments.

**Materials & Methods:**

Our work is based primarily on the clinical experience of three oncologists from the oncology department of Foch Hospital in France, who were on the frontline during the first wave of the epidemic. We compared their perceptions with published findings, to complete or nuance their views.

**Results:**

Three bio‐ethical issues were identified, and possible solutions to these problems were evaluated: (1) scientific evidence versus lack of time → the creation of emergency multidisciplinary team meetings (MTM); (2) healthcare equality versus lack of resources → the development of telemedicine; (3) individual liberties versus risk of contamination → role of cancer patients' associations, psychologists and bioethicists.

**Conclusion:**

We consider the creation of an emergency MTM, in particular, in addition to a true ethics committee with real competence in bioethics, to be a first solution that would be easy to implement in hospitals in many countries.

## 1 BACKGROUND

The emergency, intensive care and infectious disease departments of hospitals were not the only services severely affected by the first wave of the COVID‐19 pandemic.^1^ Oncology departments were also affected, albeit in a different way.^2^ Indeed, right from the start, this pandemic completely disrupted healthcare, and even research, in most of the countries affected, slowing, or even completely halting, at least temporarily, the management of cancer patients.^3^, ^4^, ^5^, ^6^ Bioethicists have been considering this topic since the start of the pandemic.^7^, ^8^, ^9^, ^10^, ^11^, ^12^, ^13^, ^14^, ^15^, ^16^ Using a specific bioethical theory and method, we try here to identify and evaluate bio‐ethical issues and possible solutions, to improve the hospital management of cancer patients during future pandemics, before the emergence of a vaccine or scientifically validated treatment.

## 2 MATERIALS AND METHODS

There are several bioethical theories and methods.^17^ The theory of the American philosophers and bioethicists Tom Beauchamp and James Childress—“Principlism”^18^—is probably the most practiced today, in various forms.^19^ However, we tend to prefer the theory of the American biochemist and bioethicist Van Rensselaer Potter—“Global bioethics”^20^—with some evolutions, for conceptual reasons explained in detail in a previous study.^21^ We consider bio‐ethical issues to be, first and foremost, tensions between moral values or standards and medical or scientific practices.^22^ The identification and evaluation of these issues and possible solutions are based on the experience of practitioners, scientific knowledge, and actual and potential effects on the quality of life—or happiness—and survival of people and societies affected by the presence, absence, or quality of these practices, taking environmental concerns and cultural differences into account.^22^ Here, we focus principally on the quality of life (i.e., happiness) and survival of cancer patients, their families and oncologists, mostly at hospital level. We sought to identify and evaluate leads, rather than to test or validate evidence.

Within this framework, we adapted an a posteriori method developed by the Canadian theologist, jurist and bioethicist Guy Durand (Table 1).^23^ Our reflection is primarily based on the clinical experience of three oncologists (=Dr. Philippe Beuzeboc, Dr. Laure Ladrat and Dr. Terence Landrin), in the broadest sense (i.e., these doctors work in an oncology department, but they are not necessarily oncologists by training), from the oncology department of Foch Hospital in France who were on the frontline during the first wave of the epidemic. We compared their perceptions with published findings, to support, complete or nuance the views of our oncologists. On 18 November 2020, these three oncologists delivered an oral presentation to the ethics committee concerning the impact of the first wave of the epidemic on the management of their cancer patients. Their oral presentations were retranscribed, in part, in a Word file report of this meeting (in French), which constituted the initial material for this study, together with scientific papers (mostly in English) identified on Google Scholar and/or PubMed, in a nonsystematic manner, with various keywords (“COVID‐19,” “cancer,” “ethics,” etc.). We consulted more than a hundred articles during the preparation of this article.

**Table 1:** A posteriori method.

Key steps
Step (1): Problem
→ Presentation of the problem, based on oncologists’ experience and/or scientific knowledge
Step (2): Issue
→ Identification of bio‐ethical issues
Step (3): Scenario
→ Evaluation of these issues in space and/or time
Step (4): Deliberation
→ Identification of possible solutions
Step (5): Perspective
→ Evaluation of these solutions in space and/or time

This study is the result of a collaboration between the ethics and oncology departments of Foch Hospital, in France. It was approved by the institutional review board of Foch Hospital (IRB 00012437).

## 3 RESULTS AND DISCUSSION

### 3.1 Problem

Should we modify our usual diagnosis and treatment protocols? The first oncologist (=Dr. Philippe Beuzeboc) to speak at the ethics committee meeting said that this was the first question he was faced with during the first wave of the epidemic in March 2020. The first issue to be addressed when trying to answer this question was the possibility that cancer might be a non‐negligible factor conferring susceptibility to a virus like SARS‐CoV‐2, or to a risk of severe forms and death from this viral infection. In the climate of doubt that prevailed shortly after the start of the pandemic, several scientific societies simultaneously emitted different recommendations to help oncologists to adapt the management of their cancer patients optimally during the epidemic.^2^, ^24^, ^25^, ^26^, ^27^, ^28^, ^29^, ^30^

However, these recommendations had almost no support from published scientific evidence specific to COVID‐19 and cancer.^31^ The almost complete absence of scientific publications at the very start of epidemic in France was the first major problem to emerge from the discourse of the first oncologist to speak. It was, therefore, impossible for the team to act in accordance with scientific knowledge validated by their peers. Unfortunately, it could not have been otherwise. This first wave that swept across France was also the first wave of this disease in most countries worldwide. China, the first country to be affected by COVID‐19, experienced its first wave only a very short time before the disease spread elsewhere.^32^ It was not until the months of May and June 2020 that the first studies were published, providing valid scientific points of reference for medical oncologists.^33^

Scientific publications have since confirmed what these oncologists (=Dr. Philippe Beuzeboc, Dr. Laure Ladrat and Dr. Terence Landrin) observed during their clinical activities at the hospital. Cancer does not seem to be a direct, significant susceptibility factor for COVID‐19.^31^ Instead, it seems to have only an indirect effect through the effects that most cancers and their treatments, particularly chemotherapy,^34^ have in cancer patients. These effects include episodes of major fatigue, and the cancer patient needing to be bedridden in a confined environment and potentially exposed to the virus for too long a period, as occurs during hospitalization, or even sometimes at home, for these cancer patients.^35^, ^36^, ^37^ Similar observations concerning the human costs of the pandemic have been made in other scientific publications.

At the end of the various first waves of the epidemic worldwide, in about June 2020, the global scientific community estimated the percentage of cancer patients infected with SARS‐CoV‐2 hospitalized due to and dying from COVID‐19 at 13%.^36^ The first of the oncologists to speak confirmed the validity of this figure in the field. Nevertheless, he also pointed out that other studies had criticized these observations and analyses, particularly for hemopathies, bronchial tumours and recent chemotherapy.^38^, ^39^, ^40^ Whatever the reason, it seems clear today that “Patients with cancer have high COVID‐19‐associated mortality rates, although there appears to be significant heterogeneity in risk among different cancer subgroups.”^41^

The second problem raised by the first oncologist to speak to the committee concerned the long‐term consequences of the healthcare priorities implemented at our hospital, and at many hospitals worldwide, favoring care continuity and adapted management in those infected with the virus. For many oncologists,^4^, ^42^ the principal fear was that, by delaying consultations for initial diagnosis or follow‐up, medical or surgical treatment, we may have considerably decreased the chances of survival in a non‐negligible number of real or potential cancer patients. There is also the problem that a number of people confined at home during lockdowns may not have wished to attend the hospital, for fear of catching and dying from COVID‐19. Studies have provided evidence in support of this hypothesis.^43^, ^44^

Indeed, we now know that, in Germany “cancer cases decreased during the first national lockdown between March 12 and April 19, 2020: by 13·9% for breast cancer, 16·5% for bladder cancer, 18·4% for gastric cancer, 19·8% for lung cancer, 22·3% for colon cancer, and 23·1% for prostate cancer,” and in the United Kingdom, “hospital admissions for chemotherapy appointments have fallen by 60%, and urgent referrals for early diagnosis of suspected cancers have decreased by 76% compared with pre‐COVID‐19 levels, which could contribute to 6270 additional deaths within 1 year.”^1^ These findings led to the prediction that “Delayed diagnosis and treatment are expected to increase the numbers of deaths up to year 5 after diagnosis by 7·9–9·6% for breast cancer, 15·3–16·6% for colorectal cancer, 4·8–5·3% for lung cancer, and 5·8–6·0% for oesophageal cancer.”^1^

At least two other problems, of a different type, were raised by the other two oncologists (=Dr. Laure Ladrat and Dr. Terence Landrin) who spoke to the committee. The first was the prohibition of visits to hospitalized relatives, because of the risk of contamination. This was particularly difficult for cancer patients nearing the end of life, or at least considered as such from a clinical and/or scientific point of view. The question of prioritization for intensive care unit places was also raised. When should cancer patients be admitted or refused? And when should palliative care be implemented? These were two major questions that these two oncologists had never before had to ask themselves, at least under such conditions. The second related problem was the difficulty experienced by families in obtaining the body of a deceased relative, particularly for cancer patients from religious cultures with specific funeral rites (with an impact on the grieving process). The potentially equally large impact on the families of cancer patients was justly recounted by these two oncologists. These points also came to the fore in published studies.^15^, ^45^, ^46^, ^47^, ^48^, ^49^, ^50^, ^51^, ^52^, ^53^

One of the major consequences for hospitals of this distress to families and cancer patients is a possible increase in rates of burnout among oncologists, which, retroactively, could have a negative effect on families and cancer patients.^54^ The European Society for Medical Oncology (ESMO) performed two online surveys. The first survey revealed that “38% of respondents stated that they had experienced feelings of burnout and 78% had felt increased concern for their personal safety since the onset of the pandemic,” whereas the second found that “the proportion of respondents reporting feelings of burnout had risen to 49%. The proportion of professionals at risk of distress increased from 25% to 33% between the two surveys.”^55^ Nevertheless, “whereas 66% of respondents in the first survey felt unable to do their job as well as they had done before the pandemic, by the time of the second survey, this proportion had decreased to 49%.*”* ^55^

### 3.2 Issues

Based on both these clinical experiences and the scientific literature, we clearly identified a first bio‐ethical issue: the inadequacy of state‐of‐the‐art practices during the first wave.^56^, ^57^, ^58^, ^59^, ^60^, ^61^ The pandemic showed the shortcomings of the dominant conception of EBM, which limits the possibilities of clinical decision‐making under conditions of uncertainty and lack of consolidated knowledge.^62^ The many benefits of EBM to patients are undeniable.^61^ Nevertheless, we can see a limitation of this approach here. How should we react to a new medical situation for which no specific scientific knowledge has yet been obtained? For many scientific, legal, moral, and ethical reasons, the studies generating such knowledge take time.^63^ We should also bear in mind that the process of scientific publication itself, outside of the particular case of so‐called “predatory” journals, also takes time.^64^ There remains a major tension between a scientific standard—“evidence,” which may be seen here as a moral standard, because of the social, and even legal sanctions imposed if it is not respected—and various new clinical practices (COVID‐19 diagnostic practices, treatments, etc.) very rapidly required in the absence of real scientific evidence (clinical trials, pharmacovigilance, etc.). This problem also concerns oncologists.

The second bio‐ethical issue identified was that of healthcare prioritization.^65^ One of the three oncologists asked the following question: by trying to save certain patients from COVID‐19 in the short term, have we not condemned others to cancer in the medium or long term? Objectively, this question appears to be legitimate, even if only at the collective scale.^66^ As we saw above, other oncologists have also posed this question, for cancer or other diseases. Can we ignore temporality during emergencies? Is there not a sort of “butterfly effect”? By modifying certain initial clinical practices, leading to the suboptimal management of a patient suffering, or potentially suffering from cancer, do we not run the risk of being responsible for other deaths? As we have already shown, a number of studies have supported this hypothesis, and the same reasoning may be applied to other diseases that are just as serious, such as various cardiovascular diseases.^1^, ^67^, ^68^, ^69^ There is, thus, a strong tension between an important moral value, “equality,” and vital clinical practices (tracheal intubation, oxygen therapies, etc.) severely limited by a real lack of material, financial and/or human resources, not only due to the pandemic but also for other reasons (the relocation of manufacturing, health policies, etc.).

A third bio‐ethical issue was identified in the psychological effects on cancer patients, families and oncologists.^52^, ^53^, ^70^, ^71^ Even in the absence of COVID‐19, a disease like cancer can have major psychological effects, not just on the cancer patients, but also on those close to them, and on the oncologist responsible for cancer patient management, particularly for paediatric cancers.^72^, ^73^, ^74^, ^75^ Anxiety, depression, and even suicide are collateral consequences of cancers that cannot be ignored, given how systemic and devastating their long‐term effects can be on the quality of life (i.e., happiness) and/or survival of the individuals concerned, whether they are cancer patients or oncologists. These effects were probably markedly exacerbated by the pandemic context.^73^, ^76^, ^77^, ^78^, ^79^ The simple limitation of family access to hospitalized and deceased patients is a finding that absolutely cannot be ignored. There is, therefore, also a major tension between another important moral value, “liberty,” and a number of clinical practices that have become highly prevalent (quarantine, containment, etc.) to limit the real risk of an increase in the global level of contamination, and its lethal and traumatic (loss of relatives, long COVID, etc.), consequences.

### 3.3 Scenario

Why are these issues so important? Let us imagine that nothing has changed and that a future pandemic of this kind occurs, for which no vaccine or other scientifically validated therapeutic solution is initially available. In such situations, oncologists will have to rely solely on clinical intuition, or on a mixture of knowledge and know‐how acquired by training and experience. Unfortunately, the “hydroxychloroquine affair” clearly demonstrated the non‐negligible limitations of this approach in terms of ethics and scientific integrity.^80^, ^81^ Priority in healthcare is still given to patients infected with the pandemic microbe, at the expense of everyone else, even when, as seen here, various clinicians and researchers highlight the possible, or even probable, deleterious consequences of this approach in the medium and long term.^1^, ^54^, ^82^ The prohibition of visits to hospitalized relatives imposed by national or regional policies and administrations would undoubtedly be repeated, even at the expense of definitively destroying the relationship of trust between oncologists and the family of the cancer patient that is so essential for effective medical practice.^83^ The outcome might be an unsatisfactory quality of life (i.e., happiness) and/or survival rate for cancer patients, as we are currently seeing.

But let us imagine an alternative scenario in which everything changes. We completely ignore clinical intuition, mainly due the “hydroxychloroquine affair,”^84^ and a certain conception of EBM.^56^ Patients suffering from the pandemic disease are not given priority because this may lead to a loss of opportunity for others, such as those with cancer, or the non‐respect of moral values and/or standards (liberty, equality, etc.) considered fundamental by some influential people.^54^, ^83^ For the same reason, we authorize all patients managed at the hospital, as for other diseases, to be accompanied by relatives.^83^ The outcome might be even more unsatisfactory in terms of quality of life (i.e., happiness) and/or survival, but in this case, for everyone, because the hospitals would probably be even more saturated.

We therefore believe that the best possible solutions to all three bio‐ethical issues would be a kind of intermediate response to the following questions (Table 2): (1) Given the initial lack of evidence and the lack of time to produce it at the start of a pandemic, how can we improve the quality of life (i.e., happiness) and/or survival of cancer patients without neglecting EBM practices? (2) Given the lack of resources for many vital clinical practices in emergency situations of this kind, how can we improve the quality of life (i.e., happiness) and/or survival of cancer patients, without neglecting healthcare equality? (3) Given the risk of contamination, how can we improve the quality of life (i.e., happiness) and/or survival of cancer patients, families and oncologists, without neglecting individual liberties?

**Table 2:** Bio‐ethical issues/questions and possible solutions/answers.

Bio‐ethical Issues & Possible Solutions
Issue #1 (question): “evidence versus lack of time?”
→ Solution (answer): “the creation of emergency multidisplinary team meetings.”
Issue #2 (question): “equality versus lack of resources?”
→ Solution (answer): “the development of telemedicine.”
Issue #3 (question): “liberty versus risk of contamination?”
→ Solution (answer): “the involvement of cancer patients' associations, psychologists and bioethicists.”

### 3.4 Deliberation

The creation of “emergency” multidisciplinary team meetings (MTM) is one possible answer to the first question (Table 2).^85^ The notion of an emergency MTM is inspired both by “classical” MTM, which are widespread in oncology departments worldwide,^86^ and the “ethics support cells” recommended by the national consultative committee for ethics in France (the CCNE).^87^ Emergency MTM would have at least two key characteristics in addition to the features of classical MTM: firstly, the collection of information and recommendations relating to the pandemic from different scientific societies and epidemiologists, and the enlargement of their multidisciplinary and interdisciplinary, or even transdisciplinary nature to the various human and social sciences. The idea is to multiply and combine scientific skills more effectively, to make up for gaps in scientifically valid clinical knowledge relating to the pandemic and, thus, to provide an alternative, temporary form of EBM guidance for oncologists in a context of considerable uncertainty, making it possible to improve the quality of life (i.e., happiness) and/or survival of cancer patients directly.

Telemedicine is a possible answer to the second question^6^, ^88^, ^89^ (Table 2). Telemedicine, which is based on information and communication technologies (ICTs), can become an essential resource during a pandemic, as already shown in a number of countries.^90^, ^91^ Indeed, various digital applications can be used to trace infected individuals and to alert those with whom they have been in contact. Other applications have made it possible for many doctors to stay in touch with their patients despite successive lockdowns and curfews.^92^ This was the case in France, where the Doctolib platform made it possible for doctors to hold consultations and to issue prescriptions remotely.^93^ It is now imperative for hospitals to develop telemedicine more extensively and to provide training in its use for their oncologists. Recent studies in the field of oncology have clearly highlighted the real and/or potential benefits of telemedicine for cancer patients in the face of COVID‐19.^94^, ^95^, ^96^, ^97^ This solution could, directly, improve the quality of life (i.e., happiness) and/or survival of cancer patients in such crises.

Psychologists and bioethicists, working with cancer patient associations, could provide an answer to the last question (Table 2). Oncologists and their cancer patients will need new medical, scientific and technical resources, but also solid human and psychological support.^15^, ^51^ Both oncologists and cancer patients can experience significant distress, as highlighted by the discourse of our three oncologists. Psychologists could provide essential psychological support for both these groups, whereas cancer patient associations could provide cancer patients with knowledge and services that emergency MTM, telemedicine or ICTs—even those with the highest level of performance —would probably be unable to offer. Bioethicists should help oncologists to reflect on their practices and should work with them to develop a new view of management, leading to new practices that could be applied during a future pandemic of this kind before the emergence of a vaccine or other scientifically validated therapeutic solution.^98^ This solution could also, directly or indirectly, improve the quality of life (i.e., happiness) and/or survival of cancer patients, their families and oncologists.

### 3.5 Perspectives

Emergency MTM could prove useful for oncologists and beneficial for cancer patients beyond the confines of our hospital. In France, under the impetus of the CCNE and other ethics committees, similar structures have emerged throughout the country since the start of the first wave of the epidemic.^99^, ^100^ A very similar tendency is also emerging in other countries.^16^, ^66^, ^101^, ^102^, ^103^ So, could a principle or general rules for the structuring, organization and functioning of such structures be developed for all hospitals, based on those conceived and implemented at our hospital? We believe that this is feasible and that the establishment of such structures is necessary, to pose questions and obtain responses from oncologists at the start of major pandemics. These structures could then actively interact with the ethics committee of the hospital to deal, more specifically, with questions of a bioethical nature, through veritable research projects in bioethics. It should nevertheless be stressed that adaptations could be made, according to the services and resources of the hospitals and the culture of the countries in which they are located.

Telemedicine is entirely dependent on ICTs, and is simply impossible without Internet connections, computer servers, computers, or even smartphones or computer tablets.^104^ These technologies require resources that are absent or of insufficient quantity or quality in many countries.^105^ This raises the question of the access of the poorest countries to new technologies that would make it possible to improve cancer patient management considerably, both within and outside the context of the COVID‐19 pandemic.^106^, ^107^ Even in countries in which these resources are available, there is a generation gap between what has been called “digital natives,”^108^ and “digital immigrants,”^109^ in other words, between those who were literally born in the digital era and those who have only really known this era as adults. For digital immigrants, access to ICTs and their use may not be easy, as shown by studies in the context of the COVID‐19 pandemic.^110^, ^111^, ^112^ These factors should be taken into account in a satisfactory bioethical deployment of telemedicine, at least in the short and medium term,^113^ especially in cancer.

Finally, based on their experiences as cancer patients, former cancer patients or relatives of cancer patients, either living or dead, the members of cancer patients’ associations are in a position to help oncologists to identify and resolve the various blind spots in their practices.^114^ However, this requires the associations to be sufficiently structured and organized for this purpose, and, indeed, to exist in the first place.^114^ In France, hospitals already work in close collaboration with such associations, and this has had a visible impact on the quality of care delivered.^115^ Nevertheless, efforts should be launched or pursued, in countries in which such associations do not exist or are insufficiently active. The same could be said for psychologists and the psychological support provided to oncologists and cancer patients, and for bioethicists and bioethical support.^98^, ^116^

An ethics committee alone, without bioethicists, would be inadequate,^117^, ^118^ because being a biologist or a physician is not sufficient, in itself, for competence in bioethics or medical ethics, just as being an oncologist does not imply intrinsic competence, for example, in orthopaedic surgery. There are courses to be followed, diplomas to be obtained, especially doctorates, and truly scientific and pedagogic experience to be acquired.^21^ It is not possible to just become a bioethicist, any more than it is possible to just become an oncologist. By a “bioethicist” we mean a researcher in bioethics, not necessarily a theologist, philosopher or lawyer by training. Bioethicists can also be physicians, biologists, veterinary surgeons, nurses or engineers.^119^ But, importantly, all bioethicists are trained academically in theories and methods; in this case to help oncologists to reflect, bioethically, on their practices, especially during future pandemics before the emergence of a vaccine or other scientifically validated therapeutic solution. In North America, and elsewhere, bioethics has developed considerably as an academic discipline in many hospitals and universities.^21^ In France, our hospital has just created its first department devoted to bioethics and composed of bioethicists, but this is far from being the case everywhere.^21^

## 4 CONCLUSION

In a future pandemic of this kind, various changes may be required in hospitals, in France and other countries to improve the quality of life (i.e., happiness) and survival of cancer patients. Through a specific bioethical theory and method, and based on both the clinical experience of three oncologists from the oncology department of our hospital and scientific publications, three bio‐ethical issues and possible solutions for improving the hospital management of cancer patients during pandemics, before the emergence of a vaccine or other scientifically validated therapeutic solution, were identified and evaluated. We consider the creation of an emergency MTM, in particular, in addition to a true ethics committee with real competence in bioethics, to be a first solution that would be easy to implement in hospitals in many countries.

## AUTHOR CONTRIBUTIONS

Henri‐Corto Stoeklé and Christian Hervé contributed equally to the writing of the manuscript. Laure Ladrat, Terence Landrin and Philippe Beuzeboc are the doctors of the departments of oncology and supportive care of Foch Hospital who agreed to participate in the study and also corrected the final manuscript.

## CONFLICT OF INTEREST

The authors declare no conflict of interest.

## Data Availability

Not applicable.
